# Forest-goers as a heterogeneous population at high-risk for malaria: a case–control study in Aceh Province, Indonesia

**DOI:** 10.1186/s12936-024-04856-8

**Published:** 2024-01-30

**Authors:** Sarah Gallalee, Iska Zarlinda, Martha G. Silaen, Chris Cotter, Carmen Cueto, Iqbal R. F. Elyazar, Jerry O. Jacobson, Roly Gosling, Michelle S. Hsiang, Adam Bennett, Farah N. Coutrier, Jennifer L. Smith

**Affiliations:** 1 Malaria Elimination Initiative, Institute for Global Health Sciences, University of California San Francisco, San Francisco, CA USA; 2 Malaria Pathogenesis Unit, Eijkman Institute for Molecular Biology, Jakarta, Indonesia; 3 Department of Women’s and Children’s Health, Uppsala University, Uppsala, Sweden; 4 Eijkman-Oxford Clinical Research Unit, Eijkman Institute for Molecular Biology, Jakarta, Indonesia; 5 Department of Disease Control, London School of Hygiene and Tropical Medicine, London, UK; 6 Department of Epidemiology and Biostatistics, University of California San Francisco, San Francisco, CA USA; 7 Department of Pediatrics, University of California San Francisco, Benioff Children’s Hospital, San Francisco, CA USA; 8 Eijkman Research Center for Molecular Biology, National Research and Innovation Agency (BRIN), Jakarta, Indonesia

**Keywords:** Malaria, Malaria elimination, High-risk populations, Surveillance, Forest-goers, Indonesia, *Plasmodium vivax*, *Plasmodium knowlesi*

## Abstract

**Background:**

A major challenge to malaria elimination is identifying and targeting populations that are harbouring residual infections and contributing to persistent transmission. In many near-elimination settings in Southeast Asia, it is known that forest-goers are at higher risk for malaria infection, but detailed information on their behaviours and exposures is not available.

**Methods:**

In Aceh Province, Indonesia, a near-elimination setting where a growing proportion of malaria is due to *Plasmodium knowlesi*, a case–control study was conducted to identify risk factors for symptomatic malaria, characteristics of forest-goers, and key intervention points. From April 2017 to September 2018, cases and controls were recruited and enrolled in a 1:3 ratio. Cases had confirmed malaria infection by rapid diagnostic test or microscopy detected at a health facility (HF). Gender-matched controls were recruited from passive case detection among individuals with suspected malaria who tested negative at a health facility (HF controls), and community-matched controls were recruited among those testing negative during active case detection. Multivariable logistic regression (unconditional for HF controls and conditional for community controls) was used to identify risk factors for symptomatic malaria infection.

**Results:**

There were 45 cases, of which 27 were *P. knowlesi*, 17 were *Plasmodium vivax*, and one was not determined. For controls, 509 and 599 participants were recruited from health facilities and the community, respectively. Forest exposures were associated with high odds of malaria; in particular, working and sleeping in the forest (HF controls: adjusted odds ratio (aOR) 21.66, 95% CI 5.09–92.26; community controls: aOR 16.78, 95% CI 2.19–128.7) and having a second residence in the forest (aOR 6.29, 95% CI 2.29–17.31 and 13.53, 95% CI 2.10–87.12). Male forest-goers were a diverse population employed in a variety of occupations including logging, farming, and mining, sleeping in settings, such as huts, tents, and barracks, and working in a wide range of group sizes. Reported use of protective measures, such as nets, hammock nets, mosquito coils, and repellents was low among forest-goers and interventions at forest residences were absent.

**Conclusions:**

Second residences in the forest and gaps in use of protective measures point to key malaria interventions to improve coverage in forest-going populations at risk for *P. knowlesi* and *P. vivax* in Aceh, Indonesia. Intensified strategies tailored to specific sub-populations will be essential to achieve elimination.

**Supplementary Information:**

The online version contains supplementary material available at 10.1186/s12936-024-04856-8.

## Background

Global achievements in reducing the burden of malaria have inspired many countries to aim for malaria elimination [1]. In countries nearing elimination, residual transmission among high-risk populations sustains transmission; however, these groups are often difficult to reach and not captured by routine malaria surveillance [2]. In many countries nearing elimination in Asia and Latin America, risk of malaria infection is linked with spending time in forests where individuals are exposed to malaria-transmitting mosquitoes [3, 4]. Identifying these risk factors, characterizing forest-going populations, and pinpointing intervention gaps are essential components of malaria elimination in these settings.

Indonesia has eliminated malaria in over half of the country’s districts and most remaining transmission occurs among forest-going populations who are difficult to identify and reach with interventions [5, 6]. In Southeast Asia, forest activities including logging, agriculture, and mining have been identified as high-risk activities with transmission occurring among adult males who have increased exposure to outdoor-biting and forest dwelling mosquitoes [7–11]. Forest workers in Indonesia are also highly mobile populations who have limited access to care and may facilitate movement of malaria between high and low burden areas [6]. The presence of the simian malaria parasite *Plasmodium knowlesi* presents additional challenges to elimination because of the zoonotic reservoir, diagnostic difficulties, differing risk factors, and a need for rigorous evaluation of interventions for this parasite species [10, 12–14].

Current regional efforts to address this high-risk population of forest-goers include research on targeted chemoprophylaxis, vector control methods to target outdoor transmission such as insecticide-treated hammock nets, assessing and improving active surveillance, and efforts to understand zoonotic crossover of simian malaria including phylogenetic analysis and evaluating ecological associations, such as deforestation [3, 6, 14, 15]. It is critical to identify additional details about subpopulations within these forest-going groups in order to tailor approaches in Indonesia and similar settings in Southeast Asia.

A case–control study was carried out in Aceh Province, Indonesia, to determine the most important risk factors for symptomatic malaria. This province has achieved reducations in malaria incidence via improved case management at health facilities, community-based bednet distribution, indoor residual spraying (IRS) and active case detection (ACD) in areas of higher endemicity; however, there has been little examination of forest activities and their associated risk in order to effectively design interventions for high-risk populations [5, 6]. The risk factors examined in this study include demographic variables, potential risk factors related to community exposures, and forest exposures (including human and simian interaction and the types and duration of activities). A subgroup analysis among forest-goers was also conducted in order to delineate high-risk sub-groups in more detail and identify key intervention points.

## Methods

### Study area

**Fig. 1:**
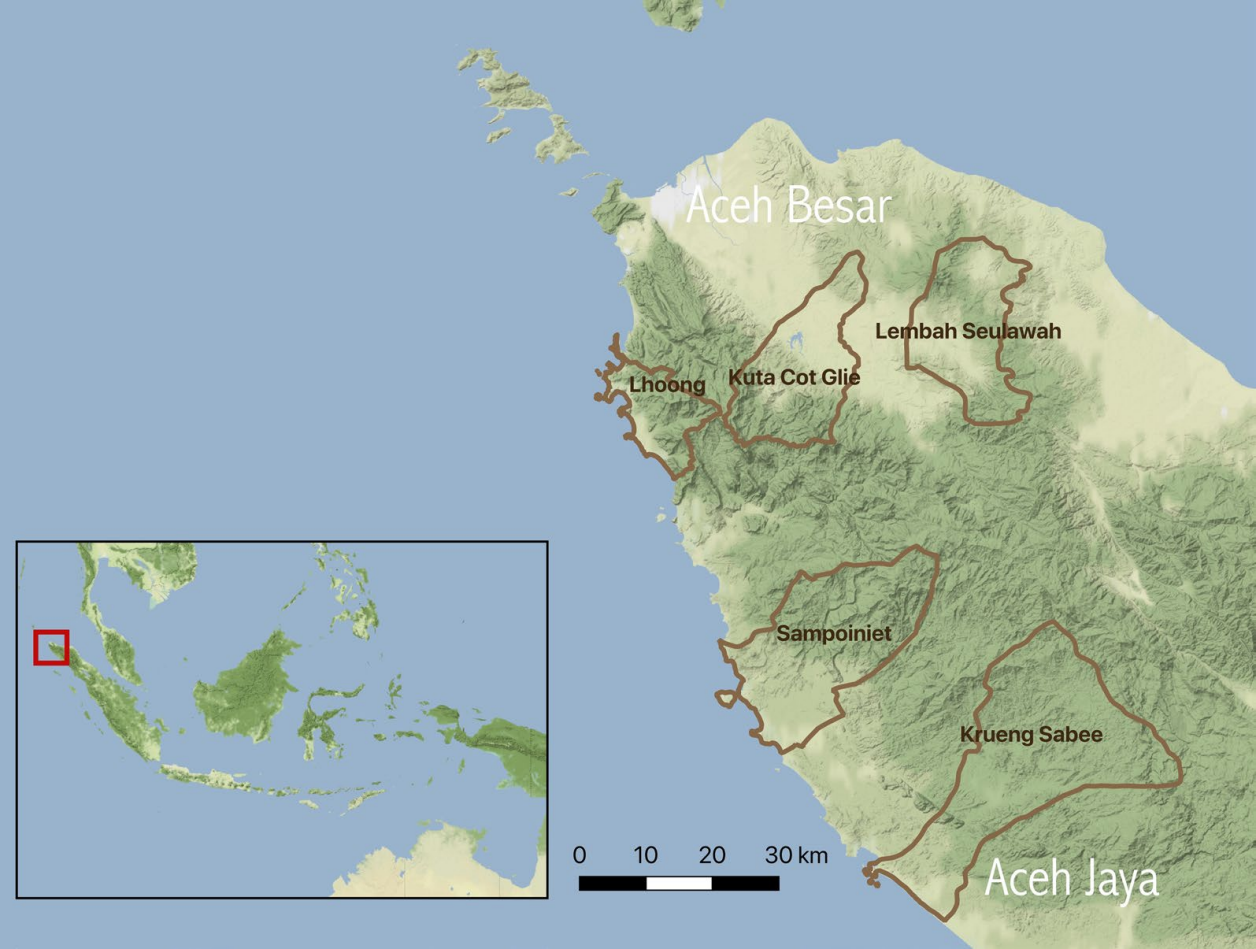
Selected study sub-districts in Aceh Besar and Aceh Jaya, Sumatra, Indonesia. Background terrain: stamen design [53]

Aceh Province is located on the island of Sumatra and is the northwesternmost province in Indonesia (Fig. 1). An estimated half of the land area in Aceh is forested and the average temperature in this region ranges from 26 °C to 28 °C [6, 16]. Major industries in Aceh Besar and Aceh Jaya include agriculture, manufacturing, mining (such as gold mining), service, and trade [6]. Malaria transmission varies across Aceh province with high malaria transmission season typically occurring from January to July [6]. Numerous malaria vectors have been documented in Aceh including *Anopheles sundaicus*, *Anopheles minimus*, *Anopheles aconitus*, and *Anopheles dirus* [17].

This study took place in five sub-district level health facility sites (Krueng Sabe, Kuta Cot Gli, Lembah Seulawah [Saree], Lhoong, and Sampoiniet) of Aceh Besar and Aceh Jaya districts (Fig. 1). Other than Sampoiniet, these were the subdistricts with the highest incidence of malaria in their district in 2016 and three of them (Kuta Cot Gli, Lhoong, Saree) were included in a study of reactive case detection (RACD) previously published [10, 12, 18]. Sampoiniet was added in July 2018 with the goal of capturing additional cases to improve statistical power. The annual parasite index (API, cases per 1000 population) among the Aceh Besar subdistricts in 2016 ranged from 0.26 and 0.50 in Saree and Lhoong, respectively to 1.44 in Kuta Cot Gli [19]. The reported study population of these three subdistricts of Aceh Besar in 2016 was 32,822 [19]. In Aceh Jaya, API was 0.29 in Sampoiniet and 4.08 in Krueng Sabe [20]. The reported study population of these two subdistricts in 2016 was 15,799 [20].

### Study design

A prospective case–control study on risk factors for symptomatic malaria was conducted in Aceh Province from April 2017 to September 2018. The study population was drawn through malaria passive case detection (patients presenting with suspected malaria to study primary care facilities or district hospitals) and active case detection (testing of largely asymptomatic individuals in the community). Patients presenting to a health facility with suspected malaria were eligible to participate as a case if they were confirmed positive for malaria by rapid diagnostic test (RDT) or microscopy and were eligible to participate as a health facility control if they tested negative. Willingness and availability to participate in the study, age of 15 or older, and residence in selected subdistricts were also inclusion criteria. All cases were captured at health facilities; additionally, polymerase chain reaction (PCR) testing and molecular testing were used to confirm positive cases and determine parasite species (further described in laboratory methods).

Two control series were utilized (health facility controls and community-based controls), allowing for the comparison of results; each of the two have particular strengths and limitations and can help compensate for any potential biases or limitations. The purpose of the health facility control series was to capture individuals who had similar selection factors to cases recruited into the study through the passive surveillance system, including a similar likelihood of reporting to a health facility for care. Age and gender are known confounders for many occupational and travel-related exposures; additionally, a formative assessment in Aceh showed that cases rarely occur in people younger than 15 and occurred more often in males [6]. In order to avoid a large imbalance and allow for the analysis of risk factors, controls were selected from RDT negative individuals aged 15 years and older and frequency matched to cases by gender.

A limitation of the test-negative design of the health facility control series is that all controls are febrile; therefore, effect estimates can be biased by any associations shared by other diseases in addition to malaria. Neighbourhood-matched community controls were used to balance this weakness. These individuals provided a population-based comparison population and were recruited from individuals who tested negative by microscopy during RACD of all subjects residing within 500 m of an index case detected in passive case detection. These controls were matched by Neighbourhood to minimize case ascertainment bias (a limitation of community controls) due to variations in geographical accessibility to the health clinics and to control for socioeconomic factors.

Original sample size calculations determined that a minimum sample size of 177 cases and 531 controls (1:3 case to control ratio) was needed to detect a difference with 80% power for the matched case–control study (correlation = 0.3). Due to declining transmission over the study period, the total number of participants recruited included 45 cases, 509 health facility controls, and 599 community controls which allows us only 45% power to detect an effect size of 2.25 and 80% power for an effect size of 3.47.

### Data collection

A survey instrument informed by formative work carried out in May–July 2016 was developed in English and translated to Bahasa Indonesia and back translated by a fluent bilingual health expert before field testing. Any interviews conducted in Acehnese were also translated into Bahasa Indonesia. The survey questions were field tested prior to data collection and administered using handheld tablets. The survey questionnaire included questions on demographic variables (subdistrict, age, gender, education, occupation, and socioeconomic status), potential community exposures to malaria at the participant’s main residence (net use, indoor residual spraying, and household building type), and forest-related exposures (sleeping in the forest, second residence in the forest, monkey sightings, and family member with forest exposure).

For health facility cases and controls, all individuals matching a recruitment profile and diagnosed in selected health facilities as confirmed positive by RDT or microscopy (cases) or confirmed negative by RDT or microscopy (controls) were interviewed by a health facility staff member on the same day. Neighbourhood controls were individuals identified during household RACD activities. These individuals were screened for inclusion, tested for malaria, and if negative by RDT or microscopy, enrolled as community controls.

### Laboratory methods

RDT and microscopy were performed in health facilities and during RACD. Quality assurance (QA) of microscopy performed at health facilities was conducted by certified microscopists at the provincial laboratory in Aceh according to national guidelines [21].

Dried blood spots (DBS) were collected using Whatman 3MM paper and sent to the Malaria Pathogenesis Laboratory at the Eijkman Institute in Jakarta. All RDT- or microscopy-positive samples (and 10% of negative samples) underwent PCR testing. Molecular testing was performed using chelex-extracted DNA from the DBS [22]. Genus-specific PCR targeting the mitochondrial *cytb* gene followed by *Alu*I enzyme digestion was used for species identification as described elsewhere [12, 23]. Additional methods were used including nPCR testing targeting the 18S rRNA gene [24] and *P. knowlesi*-specific nPCR [25]. If PCR results were not available for a case, microscopy was used to determine the species.

### Data processing

Data entry was carried out using PC and tablet computers and uploaded daily to two centralized servers. Data management, cleaning, and analysis were conducted using STATA 16.1, R 4.1.2, and QGIS 3.12.3. Six observations were dropped due to missing data for prior night net use, and four observations were dropped due to missing data for household IRS. Additionally, because there was only one case that was older than 60 years old and over one hundred controls that were over 60 years old, the dataset was restricted to participants under 60 years. Categories within variables that were conceptually similar were recombined to avoid cells smaller than five; for IRS and subdistrict, it was not possible to regroup the categories further. Potential risk factors for malaria were grouped into three broad categories: demographic variables, community exposures (at the participant’s main residence), and forest-related exposures.

Occupation was grouped into three categories: (1) farming and plantation work; (2) logging, mining, and other outdoor labour such as rattan collector, forest ranger, and elephant work; (3) other non-outdoor labour such as teaching, professional or government work, driver, or factory labour. A categorization of occupation with a larger number of groups was not possible due to small cell sizes; therefore, more detailed analyses of occupation groups were descriptive. To represent socio-economic status (SES), a principal component analysis (PCA) of household-level binary assets (e.g. electricity, radio, refrigerator, car, motor bike, cell phone, and water heater) and a categorical fuel variable was conducted [26]. To define traditional home (for primary residence), the approach by Tusting et al*.* was adapted [27] and a traditional home was defined as a building with wooden walls, partially wooden walls, or no walls. Sleeping in the forest was defined as someone who reported working in the forest or forest fringe (outside of any village) in the past 60 days and sleeping in the forest during these work trips. Additional survey questions were asked only of the forest-goers about their most recent trip to the forest. Sleeping structure in the forest was classified as tent (tent or plastic makeshift tent), hut (hut, house, or other), or barracks. The variable “used protection against mosquitoes” was defined as a binary variable with ‘yes’ indicating reported use of at least one valid method (bed net, hammock, repellent, coil, wearing covering clothes, or chemoprophylaxis).

### Data analysis

Associations between risk factors and the outcome of malaria infection were explored using unconditional logistic regression for the health facility control series and conditional logistic regression for the community control series. Model building was informed by a directed acyclic graph (DAG) conceptualizing relationships between the primary forest exposure (working and sleeping in the forest), the outcome of malaria infection, and potential confounding and mediating variables [28]. The bivariate relationships of the variables identified as potential confounders (age, gender, occupation, education, SES, net use, IRS, traditional house, and family member with forest exposure) between the main exposure and the outcome were assessed. Crude odds ratios (ORs) with 95% confidence intervals were estimated in bivariate logistic regression; variables associated with the outcome (significant at p-value 0.05) or those that changed the main effect OR by more than 10% in the final model were included.

The model building process was conducted separately for each control series. The health facility control series used an unconditional logistic regression model with fixed effects at the subdistrict level and a dummy variable for gender because the controls were frequency matched by gender (therefore ORs for subdistrict and gender are not presented). In the conditional logistic regression community control series, the community identifier was used as the grouping variable and gender was included as an a priori confounding variable. Adjusted odds ratios (aOR) and 95% confidence intervals for the variables included in the final models for each control series are presented. The SES measure was tested as a continuous and categorical measure. The second component of the PCA was included as a continuous measure for health facility controls and the first component of the PCA was included for community controls.

A descriptive subgroup analysis was conducted among forest-goers, i.e., participants who reported working and sleeping in the forest in the previous 60 days to identify risk factors specific to this group and descriptively assess the characteristics of this population. A logistic regression model (using the same model building process as above) was used to confirm key exposures using the health facility control series. The community control series was excluded from model building as it required use of the grouping variable to account for matching, which was not possible in the small subgroup. A sub-group analysis by species was not possible due to the limited number of cases.

## Results

### Descriptive analysis

**Fig. 2:**
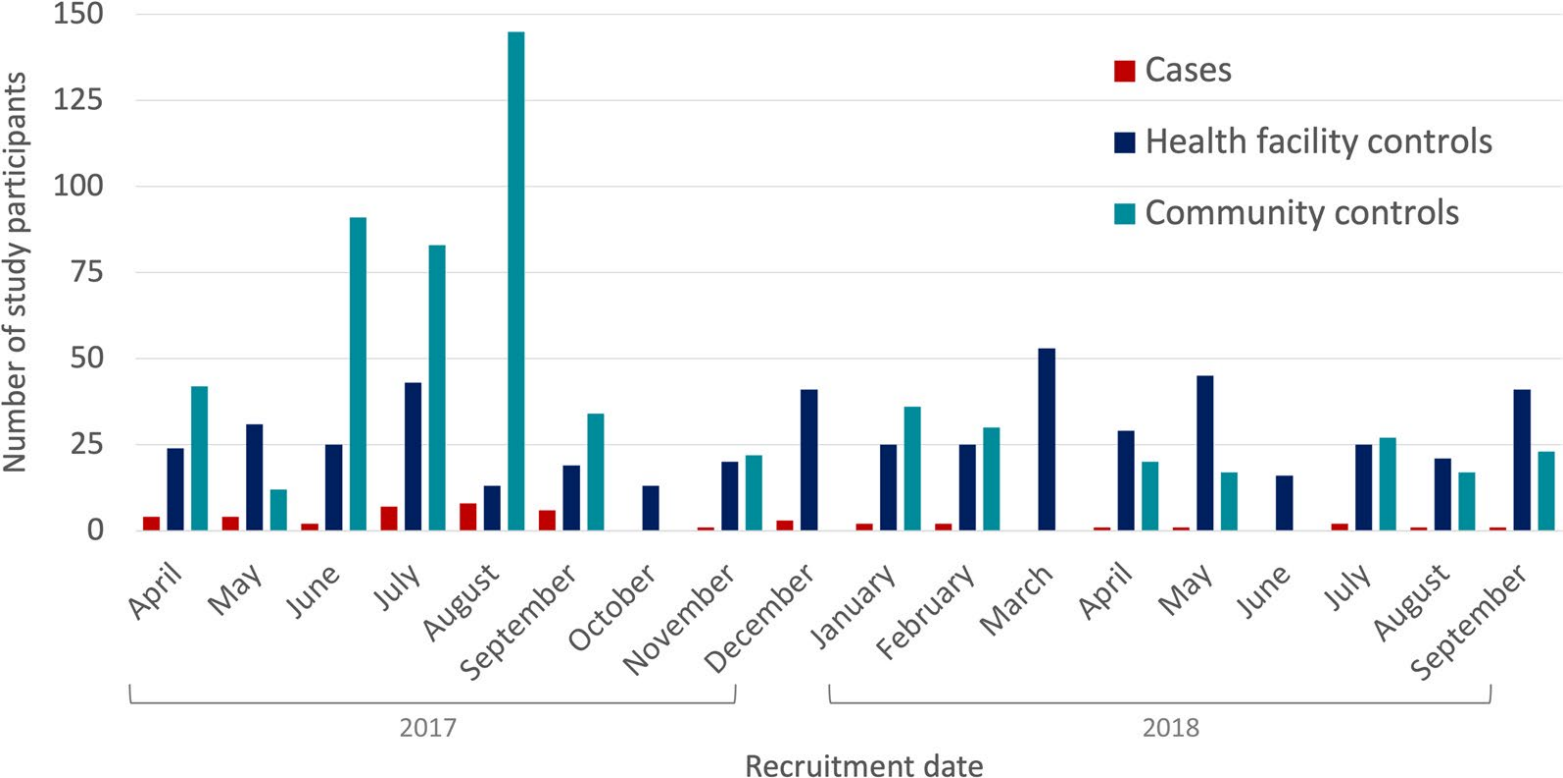
Temporal recruitment of cases, health facility controls, and community controls between April 2017 and September 2018

Between April 2017 and September 2018, 45 positive cases were identified and enrolled in the study along with 509 health facility controls and 599 community controls. Species identification by PCR was available for forty (89%) of the 45 cases, and by microscopy for the remaining cases: 27 cases (60%) were *P. knowlesi,* 17 (38%) were *Plasmodium vivax*, and for one case the species was undetermined. Case recruitment was highest during a 7 month period from April 2017–October 2017 (Fig. 2).

The distributions of demographic, community, and forest exposure information for participants are presented in Table 1. The majority of cases were male (93%) and between 30–45 years old (58%), which was similar to matched facility controls (91 and 52%) (Table 1). Community controls had a more balanced age distribution (41% male) and were similarly aged (50% between 30–45 years old). Cases were more likely to work in logging, mining, or other outdoor jobs (58%) compared to both health facility and community controls (27 and 5%) and had a similar or lower education level and SES compared to controls.

**Table 1:** Distribution of potential risk factors among index cases, health facility controls, and community controls

	Variable	Index cases	HF controls	Community controls	Total
		n = 45 (%)	n = 509 (%)	n = 599 (%)	n = 1153 (%)
Demographic variables	Age category (years)				
	15–29	9 (20)	141 (28)	201 (34)	351 (30)
	30–45	26 (58)	264 (52)	298 (50)	588 (51)
	> 45	10 (22)	104 (20)	100 (17)	214 (19)
	Male	42 (93)	461 (91)	248 (41)	751 (65)
	Post-elementary or higher education	27 (60)	370 (73)	426 (71)	823 (71)
	Occupation*				
	Farming	14 (31)	197 (39)	277 (46)	488 (42)
	Logging, mining, or other outdoor labour	26 (58)	136 (27)	27 (5)	189 (16)
	Other	5 (11)	176 (35)	295 (49)	476 (41)
	Lowest quartile SES**	13 (29)	110 (22)	175 (29)	298 (26)
	Subdistrict				
	Krueng Sabe	19 (42)	339 (67)	205 (34)	563 (49)
	Kuta Cotgli	2 (4)	36 (7)	19 (3)	57 (5)
	Lhoong	12 (27)	86 (17)	235 (39)	333 (29)
	Sampoiniet	3 (7)	9 (2)	51 (9)	63 (5)
	Saree	9 (20)	39 (8)	89 (15)	137 (12)
Community	Slept under net the previous night	23 (51)	361 (71)	317 (53)	701 (61)
	Main residence ever sprayed	4 (9)	31 (6)	115 (19)	150 (13)
	Main residence is traditional house	9 (20)	87 (17)	57 (10)	153 (13)
Forest exposures	Slept in the forest in the past 60 days	39 (87)	97 (19)	33 (6)	169 (15)
	Second residence in the forest	29 (64)	24 (5)	24 (4)	77 (7)
	Frequency of monkey sightings				
	None	20 (44)	402 (79)	340 (57)	762 (66)
	Less than once a day	15 (33)	86 (17)	200 (33)	301 (26)
	At least once a day	10 (22)	21 (4)	59 (10)	90 (8)
	Family member with forest exposure	6 (13)	9 (2)	40 (7)	55 (5)

*HF* Health Facility

^*^ “Logging, mining, or other outdoor labour” includes logger, miner, rattan collector, hole digger for phone, elephant work, forest ranger, construction; “Other” includes teacher, small business/retail, professional/technical/managerial, mechanic, government staff, military/police, driver, student, unemployed, factory labourer, domestic labour. **Socioeconomic status (SES) measure: represented as the lowest quartile of the first component of the principal component analysis (PCA). PCR speciation results were available for 40 out of 45 cases (89%)

In the category of community exposures (Table 1), cases reported a lower or similar prior night net use than health facility and community controls (51% vs. 71 and 53%). Most cases (91%) and controls (94 and 81%) reported their main residence had never been sprayed with IRS or unknown spray status. A minority of cases (20%) and controls (17 and 10%) reported living in a traditional house with wooden walls.

Forest exposures strongly differentiated case and control populations. Most cases (89% of *P. knowlesi* cases and 88% of *P. vivax* cases) reported working and sleeping in the forest in the last sixty days (Additional file 1: Table S1), compared to less than 20% of controls. Furthermore, 64% of cases reported having a second residence in the forest or forest fringe, compared to a small minority of controls (5 and 4%). Cases were also more likely to report frequent encounters with monkeys (at least once a day) and having a family member with a forest exposure in the past thirty days.

### Risk factors for malaria

Table 2 presents unadjusted and adjusted odds ratios with 95% confidence intervals for potential risk factors among health facility controls and community controls.

**Table 2:** Unadjusted and adjusted odds ratios for bivariate and multivariable unconditional logistic regression (health facility controls) and conditional logistic regression (community controls)

Variable	Health facility controls				Community controls			
	OR (95% CI)	P value	aOR (95% CI)	P value	OR (95% CI)	P value	aOR (95% CI)	P value
Demographic variables								
Gender					Female as reference			
Male	–	–	–	–	10.38 (3.09–34.85)	< 0.0001	1.25 (0.19–7.99)	0.81
Age categories								
15–29	Reference		–	–	Reference		–	–
30–45	1.50 (0.66–3.40)	0.33	–	–	1.59 (0.60–4.22)	0.35	–	–
45–59	1.33 (0.49–3.61)	0.57	–	–	1.12 (0.30–4.21)	0.87	–	–
Education								
None or elementary	Reference		Reference		Reference		Reference	
Post-elementary or higher	0.45 (0.23–0.89)	0.022	0.98 (0.40–2.40)	1.00	0.56 (0.21–1.53)	0.26	0.51 (0.08–3.45)	0.49
Occupation								
Farming	Reference		Reference		Reference		Reference	
Logging, mining, or other outdoors	4.59 (2.07–10.18)	< 0.0001	1.20 (0.41–3.53)	0.73	32.90 (8.23–131.51)	< 0.0001	12.04 (0.89–162.5)	0.061
Other	0.54 (0.18–1.63)	0.27	1.10 (0.26–4.75)	0.90	0.43 (0.12–1.50)	0.19	0.99 (0.12–8.26)	0.99
SES								
Higher SES*	0.80 (0.65–0.99)	0.044	0.76 (0.57–1.02)	0.068	1.46 (1.06–2.00)	0.020	1.66 (0.95–2.89)	0.073
Community exposures								
Net use prior night								
No	Reference		Reference		Reference		–	–
Yes	0.47 (0.24–0.92)	0.026	1.70 (0.71–4.12)	0.24	1.99 (0.70–5.68)	0.20	–	–
Household sprayed with IRS								
Never sprayed or do not know	Reference		–	–	Reference		–	–
Ever sprayed	0.61 (0.16–2.33)	0.47	–	–	0.36 (0.08–1.54)	0.17	–	–
Traditional house								
No	Reference		–	–	Reference		Reference	
Yes	1.36 (0.57–3.20)	0.49	–	–	1.55 (0.48–5.06)	0.46	0.13 (0.01–1.64)	0.120
Forest exposures								
Sleep in the forest								
No	Reference		Reference		Reference		Reference	
Yes	60.01 (17.34–207.8)	< 0.0001	21.66 (5.09–92.26)	< 0.0001	78.02 (22.68–268.4)	< 0.0001	16.78 (2.19–128.7)	0.007
Second residence in forest								
None or not in forest	Reference		Reference		Reference		Reference	
In forest/fringe	34.12 (15.61–74.53)	< 0.0001	6.29 (2.29–17.31)	< 0.0001	72.75 (20.86–253.7)	< 0.0001	13.53 (2.10–87.12)	0.006
Frequency of monkey sightings								
None	Reference		Reference		Reference		–	–
Less than once a day	3.31 (1.59–6.88)	0.001	1.19 (0.45–3.14)	0.72	1.40 (0.34–5.69)	0.64	–	–
At least once a day	8.49 (3.15–22.86)	< 0.0001	1.16 (0.32–4.15)	0.81	2.70 (0.49–14.83)	0.25	–	–
Family member with forest exposure								
No	Reference		Reference		Reference		Reference	
Yes	9.19 (2.84–29.75)	< 0.0001	4.16 (0.69–25.11)	0.12	2.53 (0.60–10.62)	0.21	0.31 (0.03–2.90)	0.31

*OR* unadjusted odds ratio, *aOR* adjusted odds ratio, *CI* confidence interval, *IRS* indoor residual spraying, *SES* Socioeconomic Status

^*^Socioeconomic measure is the second component of the principal component analysis (PCA) for health facility controls and the first component of the PCA for community controls, included as a continuous measure

#### Demographic risk factors

The adjusted odds of malaria among participants who work in logging, mining, or another outdoor job were higher than those who work in farming in both control series; this relationship was stronger and approached significance among the community control series (aOR 12.04 95% CI 0.89–162.5) (Table 2). While higher SES was associated with lower odds of malaria among the health facility controls (aOR 0.76 95% CI 0.57–1.02), higher SES was associated with higher odds of malaria among the community controls (aOR 1.66 95% CI 0.95–2.89).

#### Community exposures

The findings suggest that community exposures at the main residence of the participants were not independent risk factors for malaria after controlling for other covariates; these include net use the prior night, main residence sprayed with IRS, and living in a traditional house with wooden walls or no walls. In the unadjusted analysis using health facility controls, net use the prior night was associated with half the risk of malaria (OR 0.47 95% CI 0.24–0.92). However, this relationship did not remain present in the adjusted model nor among the community control series (Table 2).

#### Forest exposures

Forest exposures were consistently and strongly associated with risk in this population. These exposures included sleeping in the forest and having a second residence in the forest or forest fringe. Participants who reported working and sleeping in the forest in the last 60 days had 21.66 times the adjusted odds of malaria (95% CI 5.09–92.26) than those with no forest exposure, using health facility controls. Similar results were found using community controls (OR 16.78 95% CI 2.19–128.7) (Table 2). Those who reported having a second residence in the forest/forest fringe had 6.29 times the adjusted odds of malaria (95% CI 2.29–17.31) compared to those who did not have a second residence in the forest (using health facility controls; OR 13.53 95% CI 2.10–87.12 using community controls).

A higher frequency of monkey sightings and having a family member with a forest exposure were associated with higher odds of malaria in the unadjusted analysis among health facility controls, but not in the adjusted analysis with either control series.

### Subgroup analysis of forest-goers

**Fig. 3:**
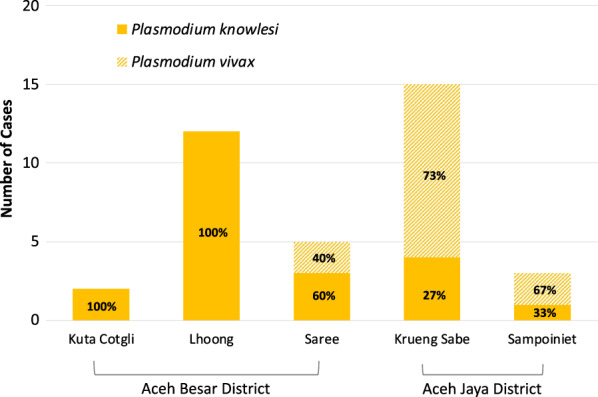
Species of malaria parasite among forest-goers by subdistrict

The subgroup analysis of forest-goers who reported working and sleeping in the forest in the past 60 days included 37 cases and 92 health facility controls (Additional file 1: Table S2), all of whom were male and the majority aged 30–45 years (57% of cases and 53% of controls). Amongst forest-going cases, 59% were *P. knowlesi* and 41% *P. vivax*. The two subdistricts in Aceh Jaya district (Krueng Sabe and Sampoinet) had a greater proportion of *P. vivax* cases, while two of the three districts in Aceh Besar had 100% *P. knowlesi* cases (Fig. 3)*.* Other characteristics in terms of education and SES were similar amongst both cases and controls. There was one key finding in the adjusted subgroup analysis: those who reported having a second residence in the forest had 8.8 times the adjusted odds (95% CI 2.4–32.3) of malaria than those who did not have a second residence in the forest (Additional file 1: Table S3). Among those who had a second residence in the forest, eighty percent reported the main reason was to be close to a work location and coverage of interventions was uniformly low (discussed below).

Occupation differed between forest-going cases and controls (p < 0.001): 51% of cases and 82% of controls worked in logging or mining while 38% and 10% worked in farming or plantation work (Additional file 1: Table 2). There were four occupations in the “other outdoor labour” category that appeared only for cases including elephant work, entomology, hole digger/rattan collector, and forest ranger. Sleeping structures differed between cases and controls (p < 0.001); the most common sleeping structure among cases was a hut (62%) and the most common sleeping structure among health facility controls was a tent (42%). Forest-going miners more commonly slept in barracks or tents while loggers reported sleeping in huts and tents. The majority (98%) of forest-goers travelled and worked in a group (Additional file 1: Table S2). The group size of forest-going farmers and loggers was generally smaller (17% of farmers reported one other worker, 65% of farmers and 83% of loggers reported 2–5 other workers) than amongst miners, in whom it was equally common for the group to be less than 5 or from 5 to 50 people.

Forest travel of both cases and controls were mainly confined to their home subdistrict (92% and 98%) although cases reported staying longer in the forest (49% and 26% over 2 weeks, p < 0.05). Forest worksite locations ranged widely and were on average 12 km away from the nearest road (min 1, max 60, median 10); overall a higher percentage of controls reported a forest location of more than 10 km from the road (p < 0.05).

Overall, there were gaps in the use of preventative measures. Seventy-six percent of forest-goers reported using at least one effective protective measure against mosquitoes during this trip; 17% used a bed net, 33% used mosquito repellent or coil, and 50% wore covering clothes (Additional file 1: Table S2). Only one person reported using a hammock net while sleeping in the forest and none of the forest-goers reported use of chemoprophylaxis. It was common to have zero bed nets at the second residence (83% of cases and 73% of controls) and 100% of forest-goers with a second residence reported that it had never been sprayed (85%) or they did not know if it had ever been sprayed (15%). The households at the second residences were primarily open construction, e.g., 69% had gaps in between the roof and walls of the structure. A higher percentage of cases (54%) than controls (17%) reported seeing monkeys at least once a day around the forest location (p < 0.001); it was also more common among *P. knowlesi* cases than *P. vivax* cases to report monkey sightings at least once a day.

## Discussion

The key findings from this study support the understanding that forest-going men are at highest risk of malaria in Aceh Province, Indonesia and provide specific details that can aid the design of tailored surveillance and response strategies targeting forest malaria. Cases had 22 times the odds of working and sleeping in the forest and six times the odds of having a second residence in the forest than controls (HF control series). Community exposures (e.g., prior night net use at main residence) were not salient risk factors; consistent with exposures that are mainly occurring in the forest. The findings in this study are relevant to other countries nearing elimination in Asia and Latin America, where forest-going (and associated activities such as mining) is often a risk factor for malaria infection [3, 4, 29, 30].

These findings are well aligned with literature in Indonesia and greater Southeast Asia that has identified high-risk populations as predominantly adult males who are exposed to outdoor biting mosquitoes via work that brings them to the forest or forest fringe for multiple days at a time [6, 11, 31]. However, there is heterogeneity within this broad group and a better understanding of subgroups and their exposure gaps will improve targeted interventions, such as tailored approaches to prevent exposures at second residences. This study identified specific characteristics of forest-going groups associated with increased risk of malaria, including having a second residence in the forest and working in occupations such as logging, mining, and farming. In addition, this study highlights the heterogeneity in forest-going populations in terms of occupation, sleeping and travel habits, worksite characteristics, and malaria parasite species, which reinforces a need for tailored intervention approaches to address forest transmission. With the potential for differing risk factors between *P. vivax* and *P. knowlesi*, gaps in the use of prevention among forest-goers, and clear diversity amongst forest-going populations, these results can help to distinguish groups and prioritize behaviours and intervention targets to be explored in designing appropriate and acceptable interventions for these high-risk populations.

A critical finding of this study is that second residences in the forest were common among forest-goers, associated with higher risk of malaria, and had a low coverage of malaria interventions. Among the full study population with both control series and in the sub-group analysis of forest-goers, those who reported having a second residence in the forest had significantly higher odds of malaria than those who did not. Anecdotal information and some limited research suggest that forest-goers in Aceh and wider Southeast Asia often sleep in makeshift or simple housing away from their primary residence [6, 11, 31]; this study provides evidence that having a second residence in the forest is a risk factor for malaria. Because participants reported that these residences have not been sprayed with IRS and there is low usage of bed nets, these second residences provide key vector control intervention targets that may help address transmission among those at highest risk of infection.

Male forest-goers in this study population were a heterogenous group: their occupations varied across farming, logging, and mining, they slept in a variety of settings including barracks, huts, or tents, and they may have worked in the forest alone or at a worksite with a large group. The majority (98%) of forest-goers travelled and worked in a group, which represents a potential opportunity to access these populations through peer networks and/or at worksites. Forest-goers were also diverse in age, level of education, and socioeconomic status. This is consistent with previous research describing the heterogeneity of forest-goers in Aceh and Southeast Asia [2, 6, 31–33].

Diverse sub-groups require tailored strategies: these findings indicate specific intervention points to target these high-risk populations and the potential to use network structure as a method to improve outreach and access. The majority of forest-goers slept in a structure such as a barrack or hut when working in the forest, and only 17% reported using bed nets (one of whom used a hammock net). Studies in other countries in Asia Pacific have found hammock nets to be acceptable to forest-goers; hammock nets should be piloted in Aceh with close attention to acceptability, feasibility, and effectiveness [15, 34]. There were large gaps in utilization of mosquito repellents and coils or wearing covering clothes (i.e., clothes that cover arms and legs). The gaps identified present intervention points where forest-goers could be targeted with forest packs with nets and repellents along with education on malaria protective measures [2, 35]. Additionally, the 37% of worksites with groups of five or more could be targeted with reactive case detection (RACD) and social and behaviour change communication (SBCC) interventions by community health workers, or screening posts at places such as transportation hubs that can provide intervention packages, malaria case management, and gather traveler information routes [2, 18]. Though RACD is widely adopted in malaria elimination settings, more information is needed on the effectiveness of RACD among forest-going populations in terms of case-finding and impact on transmission [36–38]; one study was conducted recently in Aceh to aid in answering these questions [Bennett et al., pers. commun.]. Chemoprophylaxis was not used by any forest-goers; recent studies have shown that chemoprophylaxis can be effective and acceptable for reducing malaria among forest-going populations in Southeast Asia [3, 39, 40].

Most cases in this study were *P. knowlesi,* a simian malaria parasite that was recently identified in Aceh Province by Herdiana et al*.* [41]. The descriptive findings in this study suggest that risk factors for *P. knowlesi* may differ and require specific prevention strategies than *P. vivax.* Forest-goers with *P. knowlesi* were more likely to have a second residence in the forest than those with *P. vivax*, were less likely to report using protective measures against mosquitos in the forest, and more commonly slept in huts, worked in farming, and reported sightings of monkeys. Other studies have noted that risk factors for *P. knowlesi* may differ: Grigg et al*.* found that farming occupation increased risk for *P. knowlesi* infection but not risk of other *Plasmodium* species in Malaysia [32], and Herdiana et al*.* reported species specific differences in Aceh, Indonesia [10]. A growing body of literature suggests that effective strategies to deal with this parasite species likely differ from approaches used for *P. vivax* and *P. falciparum*; for example, utilizing a one health approach in future research may be particularly useful for understanding the complexity of mosquito-borne infections with zoonotic reservoirs [13, 14, 42–44]. One of the areas in which novel tools are needed is diagnostics for *P. knowlesi*; studies have found laboratory difficulties around species distinction which can lead to misdiagnosis [12, 45], which has important implications for appropriate treatment regimens [46]. Ongoing research on vaccines for *P. knowlesi* may also provide valuable tools that can be implemented for forest goers in the future [47]. Preventing the continued rise of *P. knowlesi* will also require iterative work including the collection and incorporation of entomological surveillance data to complement epidemiologic data and inform in more detail the design of effective vector control interventions.

This study had several limitations. The first is recall bias for self-reported measures; for example, individuals who tested positive might have been more likely to remember forest-going behaviour. Second, cases were detected passively and therefore some high-risk populations who do not seek care at health facilities or malaria cases that spontaneously resolved would have been missed. Third, statistical power was limited by the small number of cases during the data collection period, leading to wide confidence intervals and a need to consolidate categories to avoid small cells. The limited cases were potentially due in part to mining and logging restrictions during the study period but also likely reflect declining transmission [6, 48, 49]. Due to this low number of cases, it was not possible to do a subgroup analysis by species. Additionally, for five cases, PCR results were not available to determine parasite species and it is also possible that some positive *P. knowlesi* cases were missed in the field due to diagnostic limitations of RDT and microscopy. The strengths of the study include two sets of control series to address different types of bias and the results with both series of controls were similar, adding to the evidence to support the conclusions of this study. Implementation via routine surveillance was another strength: the study was conducted through the provincial national malaria programme in Aceh Province, including the subdistrict health facilities and health facility staff.

The final persistent pockets of transmission among high-risk populations will be the hardest to eliminate [3, 5]. In this study, malaria risk factors are identified in Aceh Indonesia that represent exposure gaps and intervention targets that should inform collaborative design and evaluation of acceptable interventions in an iterative fashion to halt transmission [50]. Malaria continues to burden the most vulnerable populations in Indonesia and globally; working with local communities to address malaria among high-risk populations via effective interventions is essential to global equity [51, 52].

## Conclusions

Malaria high-risk populations in Aceh, Indonesia are predominantly forest-goers who are a diverse population; the findings of this study delineate sub-groups among forest-goers and outline gaps to be targeted with interventions. Cases had higher odds of working and sleeping in the forest and having a second residence in the forest than controls. The recommended interventions target second residences in the forest and aim to complement forest-going behaviour. This study also highlights the threat of *P. knowlesi*, which has increased in priority and threatens to reverse many years of progress towards malaria elimination in Indonesia and Southeast Asia. Additional research on effective methods for preventing and mitigating this parasite species among forest-goers will be a key challenge. Rigorous targeting of effective interventions to these populations is essential to achieve malaria elimination in Indonesia and other settings challenged by forest malaria.

### Supplementary Information

**Additional file 1: Table S1**. Distribution of demographic variables of cases by species. *Socioeconomic status (SES) measure: represented as the lowest quartile of the first component of the principal analysis.

**Additional file 2: Table S2. **Distribution of potential risk factors among forest-goers. *Socioeconomic status (SES) measure: represented as the lowest quartile of the first component of the principal component analysis (PCA). **Occupation categories grouped differently than in main analysis (all occupations were outdoor occupations).

**Additional file 3 : Table S3. **Unadjusted and adjusted odds ratios for bivariate and multivariable unconditional logistic regression (health facility controls) among subgroup analysis of forest-goers. OR unadjusted odds ratio, aOR adjusted odds ratio, CI confidence interval. *Occupation categories grouped differently than in main. “Other outdoor occupation” includes driving, elephant work, entomology, field technician, forest ranger, hole digging, hunting, rattan collecting **Socioeconomic measure is the second component of the principal component analysis (PCA), included as a continuous measure. Note: individual protective measures (coil, bed net, covering clothes) not included due to collinearity with use of protective measures (and none were significant in bivariate regression). The following variables were not included due to small cells: sex (all forest-goers were male), mosquitoes present, and trip destination.

## Data Availability

Data used for this manuscript are available upon request.

## Abbreviations

aOR: Adjusted odds ratio
ACD: Active case detection
CI: Confidence interval
DAG: Directed acyclic graph
HF: Health facility
IRS: Indoor residual spraying
OR: Odds ratio
SBCC: Social and behaviour change communication
SES: Socio-economic Status
PCA: Principal component analysis
PCR: Polymerase chain reaction
RACD: Reactive case detection
RDT: Rapid diagnostic test
